# Design and conduct of the activated protein C and corticosteroids for human septic shock (APROCCHSS) trial

**DOI:** 10.1186/s13613-016-0147-3

**Published:** 2016-05-06

**Authors:** Djillali Annane, Christian Brun Buisson, Alain Cariou, Claude Martin, Benoit Misset, Alain Renault, Blandine Lehmann, Valérie Millul, Virginie Maxime, Eric Bellissant, The APROCCHSS Investigators for the TRIGGERSEP Network

**Affiliations:** 1 General ICU, Service de Réanimation, Hôpital Raymond Poincaré, Laboratory of Infection and Inflammation, U1173, AP-HP, University of Versailles SQY and INSERM, 104 Boulevard Raymond Poincaré, 92380 Garches, France; 2 Service de Réanimation Médicale, Hôpital Henri Mondor, AP-HP, Créteil, France; 3 Service de Réanimation Médicale, Hôpital Cochin, AP-HP, Paris, France; 4 Service d’Anesthésie Réanimation, Hôpital Nord, AP-HM, Marseille, France; 5 Service de Médecine Intensive et Réanimation, Hôpital Saint-Joseph, AP-HP, Université Paris Descartes, Paris Sorbonne Cité, Paris, France; 6 Service de Pharmacologie, Centre d’Investigation Clinique INSERM 1414, CHU de Rennes, Université de Rennes 1, Rennes, France; 7 Département des Essais Cliniques, AGEPS, Paris, France; 8 Délégation à la Recherche Clinique, Hôpital Saint-Louis, AP-HP, Paris, France

**Keywords:** Septic shock, Clinical trial, Design, Ethics, Corticosteroids

## Abstract

**Background:**

We aimed at assessing the benefit-to-risk ratio of activated protein C (drotrecogin-alfa activated, DAA) and corticosteroids, given alone or in combination, in patients with septic shock.

**Methods:**

We implemented an investigator-led, publicly funded, multicenter, randomized according to a 2 × 2 factorial design, placebo-controlled, double-blind trial in four parallel groups in which adults with persistent septic shock and no contraindication to DAA were assigned to either DAA alone (24 mg/kg/h for 96 h), or hydrocortisone (50 mg intravenous bolus q6 for 7 days) and fludrocortisone (50 µg once daily through the nasogastric tube for 7 days) alone, or their respective combinations, or their respective placebos. Primary endpoint was 90-day mortality rate. Follow-up duration was 6 months. Statistical analysis was planned to be performed in intent-to-treat once after all participants completed 180-day follow-up and according to the 2 × 2 factorial design.

**Results:**

The first patient was recruited in September 2008. The trial was suspended on October 25, 2011, owing to the withdrawal from the market of DAA. At this time, 411 patients had been enrolled. On May 17, 2012, the continuation of the trial on two parallel groups was approved by all legal authorities with the aim of investigating the benefit-to-risk ratio of corticosteroids. On June 30, 2014, the trial was suspended again by the study sponsor upon request of the independent data and safety monitoring board. Recruitment restarted on October 7, 2014, after any safety concern was ruled out. Finally, the trial was completed on June 23, 2015, with the recruitment of 1241 patients.

**Conclusions:**

This report details the design, statistical plan and conduct of a randomized controlled trial of hydrocortisone and fludrocortisone in septic shock.

*Trial registration* The trial was registered at ClinicalTrials.gov under NCT00625209

## Background

Septic shock is now defined by a vasopressor requirement to maintain a mean arterial pressure ≥65 mmHg and serum lactate >2 mmol/l in the absence of hypovolemia [1] (it was defined by the need for vasopressors to restore cardiovascular homeostasis [2] at the time the protocol was planned). Its mortality and sequalae remain unacceptably high with roughly one out of two patients dying within 1 year and half of survivors suffering from cognitive decline [3]. International guidelines recommend immediate source control and antibiotics, fluid resuscitation and norepinephrine [4].

 Drotrecogin-alfa activated (DAA) was the only marketed therapy for sepsis after promising early findings [5]. Subsequent investigations failed to reproduce survival benefit from this drug in sepsis [6] or in septic shock [7, 8].

Corticosteroids have been used for more than 60 years, mainly in inflammatory conditions, autoimmune diseases or cancer. They have also been used in infectious diseases and sepsis. Corticosteroids have pleiotropic effects including immune modulation, metabolic and cardiovascular effects. So far, sepsis trials have failed to rule in or out survival benefit from corticosteroids. Most physicians and researchers would agree against using short course (<3 days) of high dose (>400 mg per day of hydrocortisone or equivalent) in sepsis [4]. Treatment with ≤400 mg per day of hydrocortisone (or equivalent) for ≥3 days has variably been associated with survival benefit [9]. The two largest trials of corticosteroids for septic shock with 300 [10] and 500 [11] patients, respectively, reported treatment benefits in terms of hemodynamics and organ functions. One trial showed survival benefit in septic shock and blunted cortisol response to corticotrophin [10]. There were remarkable differences in trials’ design (Table 1). Current guidelines suggest restricting hydrocortisone to patients who are poorly responsive to vasopressors, not using fludrocortisone and not based on Synacthen test [4].

**Table 1:** Comparison of (a) targeted population, (b) experimental treatments of four trials on corticosteroids for septic shock

	Ger-Inf-05	CORTICUS	APROCCHSS	ADRENALS
(a)				
Expected sample size	300	800	1240	3800
Actual sample size	300	500	1241	?
Time window for inclusion since onset of shock	3 h then protocol amended for 8 h	72 h	24 h	24 h
Age	≥18 years	≥18 years	≥18 years	≥18 years
Shock criteria	SBP < 90 mm Hg for at least 1 h despite adequate fluid replacement and >5 μg/kg/h of dopamine or epinephrine or norepinephrine; Arterial lactate >2 mmol/l	SBP < 90 mmHg or decrease >50 mmHg in SBP in previous hypertensive patients despite adequate fluid replacement or need for vasopressors to maintain SBP ≥ 90 mmHg Administration of vasopressor for ≥1 h	Norepinephrine or epinephrine at a rate ≥0.25 µg/kg/min or ≥1 mg/h) or any other vasopressor to maintain SBP ≥ 90 mmHg or MBP ≥ 65 mmHg Administration of vasopressors for ≥6 h	Vasopressors or inotropes to maintain a SBP > 90 mmHg, or MBP > 60 mmHg, or a MBP target set by the treating clinician for maintaining perfusion Administration of vasopressors or inotropes for =4 h
Mechanical ventilation as a mandatory entry criteria	Yes	No	No	Yes
Organ failure	Urinary output of <0.5 ml/kg for ≥1 h Or PaO_2_/FIO_2_ < 280 mmHg	Urine output <0.5 ml/kg/h for ≥1 h Or pH < 7.3, or arterial base deficit ≥5.0 mmol/l, or arterial lactate >2 mmol/l Or PaO_2_/FIO_2_ < 280 in the absence of pneumonia, and <200 in the presence of pneumonia Or platelet count ≤100,000 cells/mm^3^ Or Glasgow Coma Scale <14 or acute change from baseline)	Sequential Organ Failure Assessment (SOFA) score ≥3 for ≥2 organs for ≥6 consecutive hours	Not mentioned
Non-responders to the Synacthen test as the primary subgroup of interest	Yes	Yes	Yes	No
(b)				
Type of corticosteroids	Hydrocortisone hemisuccinate and 9-α-fludrocortisone	Hydrocortisone hemisuccinate	Hydrocortisone hemisuccinate and 9-α-fludrocortisone	Hydrocortisone
Dose per day	Hydrocortisone 200 mg Fludrocortisone 50 µg	200 mg	Hydrocortisone 200 mg Fludrocortisone 50 µg	200 mg
Route of administration	Hydrocortisone: four intravenous bolus of 50 mg Fludrocortisone: 50 µg via the nasogastric tube	Four intravenous bolus of 50 mg	Hydrocortisone: four intravenous bolus of 50 mg Fludrocortisone: 50 µg via the nasogastric tube	Intravenous continuous infusion rate without loading dose
Duration	Seven days at full dose	Five days at full dose then tapered to 50 mg intravenously every 12 h for days 6 to 8, 50 mg every 24 h for days 9 to 11, and then stopped	Seven days at full dose	Seven days at full dose, while in the ICU

## Description of the study design

### Clinical question and trial objectives

On the one hand, there was a strong rationale for using DAA or corticosteroids in sepsis [12]. DAA may act via direct immunomodulation and by counteracting sepsis-induced hypercoagulopathy [12, 13]. It was associated with survival benefit in a phase III trial in severe sepsis [5]. The hypothalamic–pituitary–adrenal (HPA) axis was identified as the main endogenous counter-regulator of inflammation [14]. Then, disrupted HPA axis and exaggerated inflammation may favor multiple organ failure and death in sepsis [15]. Corticosteroids were associated in Ger-Inf-05 [10], a phase III trial of hydrocortisone plus fludrocortisone, with survival benefit in septic shock and post-corticotrophin increase in cortisol levels ≤9 µg/dl, so-called non-responders [16].

On the other hand, there was equipoise among physicians about routine use of DAA and corticosteroids [17, 18]. Although DAA was approved for sepsis, failure to confirm its benefit in mild sepsis [6] yielded controversy about its benefits and risks with limited use in routine practice. Likewise, CORTICUS cast doubt about the benefit of hydrocortisone [11].

Therefore, the activated protein C and corticosteroids for human septic shock (APROCCHSS) trial aimed at evaluating in septic shock the benefit-to-risk ratio of DAA and corticosteroids, given alone or in combination, and any interaction between response to corticosteroids and non-responder status.

### Study design

We designed an investigator-led, publicly funded, multicenter, randomized, 2 × 2 factorial, placebo-controlled, double-blind trial in four parallel groups.

### Selection of study population

Evidence suggested greater benefit from DAA or corticosteroids in septic shock than in sepsis, in adults than in children, and when administered within 24 h [12]. In routine practice, physicians may consider adjunct therapy after optimal management of sepsis source and of organs function [2].

Therefore, were eligible in the trial, adults admitted to the intensive care unit (ICU) for <7 days and with indisputable or probable septic shock [2] for <24 h. Septic shock was defined by (1) clinically or microbiologically documented infection, (2) Sequential Organ Failure Assessment (SOFA) [19] score ≥3 for ≥2 organs for ≥6 consecutive hours, (3) treatment for ≥6 h with catecholamines (≥0.25 µg/kg/min or ≥1 mg/h of norepinephrine, epinephrine or any other vasopressor) to maintain systolic (SBP) ≥90 mmHg or mean blood pressure (MBP) ≥65 mmHg. Patient’s informed consent or next of kin assent was obtained before inclusion whenever possible. Otherwise, deferred consent from patients was recorded [20].

Owing to the risk of bleeding with DAA [21], non-inclusion criteria were (1) surgical procedure ≤7 days; (2) gastrointestinal bleeding ≤6 weeks; (3) chronic liver disease; (4) trauma ≤3 months; (5) any intracranial mass, stroke or head injury ≤3 months; (6) thrombocytopenia <30,000/mm^3^; (7) formal indication for anticoagulation except venous thromboembolism prophylaxis which should be continued whenever indicated; (8) any other condition with increased risk of bleeding, as per patient’s physician. Owing to uncertainty of effects of DAA or corticosteroids in pregnant women and in newborns, patients were not included in case of pregnancy or lactation. Additional non-inclusion criteria were (1) patients with palliative goals, (2) underlying fatal (≤1 month) condition, (3) patients currently taking corticosteroids (30 mg prednisone equivalent ≥1 month), (4) known hypersensitivity to DAA, (5) no affiliation to social security.

A priori defined subgroups of interest to explore survival benefits from corticosteroids were: (1) non-responders, i.e., patients who increase their cortisol levels by 9 µg/dl or less at 30 and 60 min following 250 µg intravenous bolus of corticotrophin; (2) community-acquired pneumonia; (3) acute respiratory distress syndrome (ARDS). Indeed, it was expected that non-responders, patients with community-acquired pneumonia or ARDS may be more likely to draw survival benefit from corticosteroids [9].

### Study interventions

DAA was infused intravenously at 24 μg/kg/h from foil-wrapped bags for 96 h. Infusion was interrupted 2 h before any percutaneous procedure or major surgery and resumed 1 or 12 h later, respectively, in the absence of bleeding complication. Identical blinded volume of 0.9 % saline was used as placebo for DAA.

Type, dose and duration (7 days) of corticosteroids were determined according to Ger-Inf-05 [10] and not to CORTICUS [11]. Hydrocortisone was administered as 50 mg intravenous bolus every 6 h, and 50 µg tablet of fludrocortisone was given via the nasogastric tube once daily in the morning. Placebos of French commercial forms of hydrocortisone and fludrocortisone were manufactured for the requirements of the trial. Active and placebo drugs had similar aspects (checked and certified by qualified persons for each batch), i.e., vials of white, freeze-dried powder for parenteral use of hydrocortisone hemisuccinate 100 mg or placebo, and tablets for oral fludrocortisone 50 µg or placebo in blisters of ten.

Co-interventions were harmonized across centers according to 2008 Surviving Sepsis Campaign guidelines [22]. Blood glucose levels were monitored at least every 4 h and maintained at ≤150 mg/dl by intravenous infusion of insulin. Intravenous broad-spectrum antibiotics were given after drawing specimen from all potential sites of infection and adjusted to actual pathogens whenever needed. Investigators followed guidelines for the prevention of superinfection [23]. Open-labeled corticosteroids and non-steroidal anti-inflammatory drugs, open-labeled DAA, anti-thrombin III and any anticoagulant (except venous thromboembolism prophylaxis) were discouraged. The use of antiplatelet agents was left at physicians’ discretion. Neuromuscular blockade agents were discouraged, except in the first 24 h of refractory hypoxia. The investigators were provided with published guidelines for the management of septic shock. We systematically recorded, on a daily basis during the first week post-randomization, in the case report form, blood glucose levels and dose and duration of insulin, source and pathogens identification and dose and duration of any antibiotic administered to the patients, type and dose of fluid therapy and of vasopressor therapy, score of sedation and delirium and use of any sedation or neuromuscular blocking drug, dose and duration of any anticoagulant, and type, dose and reason for use of any open-labeled corticosteroids. These data will allow careful examination of the adherence of investigators to guidelines for co-interventions. In addition, at each investigators meeting, these guidelines were systematically discussed to reinforce compliance.

### Randomization and blinding

Randomization was centralized, through a secured Web site, and stratified according to center, using permutation blocks where size was unknown by investigators. Patients were randomly allocated to receive DAA and hydrocortisone plus fludrocortisone, placebo of DAA and hydrocortisone plus fludrocortisone, or DAA and placebos of hydrocortisone and fludrocortisone, or all placebos. The day of randomization was considered as study day 0. The system printed prefilled prescriptions and, during the first part of the trial, individual infusion tables of DAA/placebo for care unit and pharmacy.

Treatment boxes were coded and masked centrally. Corticosteroids or placebos were sealed in sequentially numbered, identical boxes containing 30 vials of lyophilized hydrocortisone and 30 ampoules of injectable water, and a blister package with 10 tablets of fludrocortisone or its placebo. Each box has a detachable sticker for traceability of dispensing by hospital pharmacy and for administration by nurses.

Numbered boxes of DAA/placebo were also prepared on site. One patient received one corticosteroid/placebo box and one DAA/placebo box with the same randomization number. All study drugs were shipped to participating sites by AGEPS, AP-HP, Paris, France. The sequence was concealed from patients, staff members, investigators, members of independent data safety monitoring board (DSMB), sponsor and local pharmacists for corticosteroids.

### Definitions

*Organ system failure* was defined for each of the 6 major organ systems as a SOFA score of 3 or 4 points (on a scale of 0–4 for each organ system, for an aggregate score of 0–24, with higher scores indicating more severe organ dysfunction) [19]. *Reversal of shock* was defined as MBP ≥ 60 mmHg for ≥24 h after cessation of vasopressor. *Superinfection* was defined using standard criteria [23], as a new infection occurring ≥48 h after randomization. *New sepsis* was defined as a new episode with microbiologic confirmation. *New septic shock* was defined as a new episode of septic shock after reversal of the initial episode.

### Investigated parameters and follow-up

#### Baseline data

We systematically recorded (1) demographic and anthropometric data; (2) time of hospital and ICU admissions; (3) location prior to ICU admission (community, hospital, long-term care facility); (4) co-morbidities using Acute Physiology and Chronic Health Evaluation (APACHE) disability scale [24] and McCabe class [25]; (5) severity of illness using vital signs, Simplified Acute Physiology Score (SAPS) II [26] and SAPS III [27] and SOFA score [19]; (6) core temperature; (7) type and dose of any antibiotics in the week before randomization; (8) type and dose of vasopressors and inotropic drugs; (9) time from shock onset; (10) hematologic, chemical data, blood gas analyses and arterial lactate levels; (11) Gram examination and cultures of samples from any suspected site of infection; (12) plasma cortisol levels before, 30 and 60 min after 250 µg intravenous bolus of corticotrophin.

#### Follow-up

Patients were followed up for 180 days. We recorded daily from randomization to study day 7, at day 10, day 14, day 21 and day 28 or at ICU discharge (depending on which occurred first): (1) vital signs; (2) muscular disability rating score (MDRS) ranging from 1 to 5 with 1 meaning no deficit, 2 minimal deficit or atrophy, 3 mild-to-moderate distal deficit, 4 mild-to-moderate proximal deficit and 5 severe proximal deficit or atrophy [28]; (3) CAM-ICU scale [29]; (4) any bleeding; (5) results from standard laboratory tests; (6) cultures of specimens from any new suspected site of infection; (7) cumulated doses of intravenous insulin (UI/L/24 h), minimal and maximal infusion rates (µg/h; given for at least 1 h) of catecholamine, blood product transfusion, mode of ventilation (spontaneous breathing, noninvasive or invasive mechanical ventilation), need for and mode of renal replacement therapy and treatment with statins; (8) SOFA scores. If the patient was sedated, MDRS score and CAM-ICU were assessed ≥6 h after interruption of sedation. In case of abnormal neurological status, brain imaging (CT scan or magnetic resonance imaging) was performed.

Mid-term sequalae were assessed at day 90 and day 180 post-randomization, by means of the Short-Form General Health Survey [30], the Impact of Events scale [31] and Hospital Anxiety and Depression scale [32].

### Biobanking

Fifteen milliliters of blood was sampled at baseline, day 1, day 4 and day 7. Then, aliquots of serum and plasma were stored at −80 °C, and DNA was stored at +4 °C at each participating hospital. On a regularly basis, samples were shipped to a core laboratory.

### Study endpoints

Ninety-day mortality was the primary endpoint. Although most of sepsis-related deaths occur within 28 days, increasing evidence suggests that sepsis continues to kill patients beyond day 28. In previous sepsis trials, significant number of 28-day survivors died during the same hospital stay [33].

 Secondary endpoints included (1) death rates at ICU and hospital discharge, at day 28 and day 180; (2) proportion of patients with decision to withhold/withdraw care; (3) time to wean off vasopressors, i.e., shock reversal; (4) number of days alive (up to 28 and 90 days) and free of vasopressors; (5) time to SOFA score <6; (6) number of days alive (up to 28 and 90 days) and with SOFA score <6; (7) time to wean off mechanical ventilation; (8) number of days alive (up to 28 and 90 days) and free of mechanical ventilation; (9) length of ICU and hospital stay in all patients and in survivors.

Safety outcomes included occurrence up to 90 days of superinfection, new sepsis, new septic shock, gastrointestinal bleeding and neurological sequalae (cognitive impairment and muscles weakness) at ICU and hospital discharge, at day 90 and at day 180.

### Sample size calculation and statistical analysis plan

We anticipated a 90-day mortality rate among patients with septic shock of 45 % [10]. Using 2 × 2 factorial design with a bilateral formulation, 320 patients per group (i.e., total of 1280 patients) were needed to detect an absolute reduction of 10 % of 90-day mortality (*α* = 0.05 and power at 95 %) with either DAA or corticosteroids.

After DAA withdrawal from the market, we analyzed the effects of DAA in patients included before trial suspension without analyzing the effects of corticosteroids [8].

As initially planned in the protocol, statistical analyses will be performed in intent-to-treat after all participants have completed 180-day follow-up and according to the 2 × 2 factorial design. This analysis will allow assessing the interaction between DAA and corticosteroids and corticosteroids effect (on the basis of a comparison of 2 groups of approximately 620 patients) with the initially planned power. The effect of DAA will be reanalyzed on the basis of a comparison including 208 patients with DAA and 1033 patients without DAA with a statistical power of 76 %.

For continuous variables, means and SD or median (IQR), in case of non-normality of distribution, will be reported. For categorical variables, number of patients in each category and corresponding percentages will be given. Missing data will not be replaced.

The effects of treatments on frequency of fatal events (mortality rates at day 28, at day 90, at discharge from ICU or from hospital and at day 180) will be compared using a logistic regression. The same analysis will be used for the proportion of patients with decision to withhold/withdraw care and safety outcomes. An analysis of variance will be used to compare continuous variables as length of stay. Cumulative event curves (censored endpoints) will be estimated with Kaplan–Meier procedure, and Cox model will be used to compare treatments effects (time to death, time to wean off vasopressors and mechanical ventilation, time to a SOFA score <6). Analysis of variance will be used to compare number of days alive and free of vasopressors, mechanical ventilation, and with a SOFA score <6.

The same analyses will be conducted for subgroups unless the numbers of patients are insufficient. In this case, statistical methods will be adapted according to sample sizes.

The statistical analysis plan will be revised after blind review of data and before access to randomization list. All analyses will be conducted with SAS statistical software (version 9.4; Cary, NC, USA).

### Study Organization and Funding

The protocol was approved by all investigators on June 2007. It was independently approved for scientific and financial aspects by the national jury of the Clinical Research Hospital Program on October 2007, and the Ministry of Health confirmed funding under contract number P 070128. The protocol and qualification of all investigators were approved by the Ethics Committee (*Comité de Protection des Personnes*, *CPP*) of Saint-Germain-en-Laye, France, on November 22, 2007. The CPP allowed for waiver of consent and deferred consent.

DSMB was set up prior to recruitment of the first patient, included experts in critical care medicine, infectious diseases, pharmacology and statistics, had full access to raw data and met on a regular basis.

Data monitoring was performed by the sponsor (*AP*-*HP; Délégation à la Recherche Clinique d’Ile de France, DRRC*). AP-HP had full access to patients’ charts and checked all data recorded onto the electronic case report form (CRF) against original chart. The trial used a Web-based electronic CRF (Telemedecine Technology, France).

Data management and statistical analysis were performed independently of the sponsor and of investigators by specialized Biometry unit (*Unité de Biométrie, INSERM 1414 Clinical Investigation Centre, Rennes University Hospital, Rennes 1 University, Rennes France*).

Institutional pharmacists (AGEPS) were responsible for obtaining corticosteroids and their placebos, shipping study drugs to participating sites and getting back unused drugs. They were responsible for accurateness of blinding and pharmaceutical organization of the trial.

Vials of lyophilizate for parenteral use of hydrocortisone hemisuccinate 100 mg or placebo and diluent ampoules were obtained from Serb pharmaceutical company. Active and placebo tablets for oral route of fludrocortisone 50 µg were supplied from French commercial market under responsibility of AGEPS. Due to the study length, fludrocortisone shelf-life and commercial changes, different companies were involved: EP-HP AP-HP, Genopharm and HAC pharma company. No change in formulation, specifications or quality controls occurred. This was approved by National Agency for Drug Safety (ANSM, Saint-Denis, France). Anticipation of manufacturing campaigns avoided any supply disruption.

Several documents were provided to caregivers and pharmacists to ensure safety, compliance and good use of study drugs: prefilled prescriptions (with patient ID, weight, treatment number, DAA/placebo infusions description when required), DAA/placebo personalized administration table (number of perfusions, dose/perfusion, durations, etc.), DAA/placebo preparation sheet for hospital pharmacists, nurse tracking administration file, pharmacy dispensing and return book. All documents were revised after DAA withdrawal from the market.

A total of 65 ICU were authorized to recruit patients, 19 at university hospitals and 46 at community hospitals. Among them 34 participated actively in recruiting patients in this trial.

### Trial registration

The trial was registered on February 19, 2008, before inclusion of the first patient, at ClinicalTrials.gov under the number NCT00625209.

## Study conduct

### Study suspension and completion

The first patient was recruited on September 2, 2008, and the last patient on June 23, 2015. The study was suspended twice. Study sponsor, Steering committee, investigators, pharmacists and study statisticians always remained blinded to study treatments.

On October 25, 2011, while the trial was still recruiting, Lilly announced DAA withdrawal from the market [34]. Since DAA was no longer available and Lilly refused to provide the drug for our study, AP-HP immediately suspended the study. The Steering committee, while remaining blinded to study treatments, proposed to analyze effects of DAA in the 411 patients included before trial suspension without analyzing effects of corticosteroids as there was no interaction between the two treatments. There was no benefit from DAA [8]. The Steering committee recommended trial’s continuation to assess effects of corticosteroids. These recommendations were approved by ethics committee on February 10, 2012, and by ANSM on April 18, 2012. Inclusions into the study restarted on May 18, 2012. On June 24, 2013, additional funding was granted from Ministry of Health (contract P 12-002-0030; AOM 07008).

**Fig. 1:**
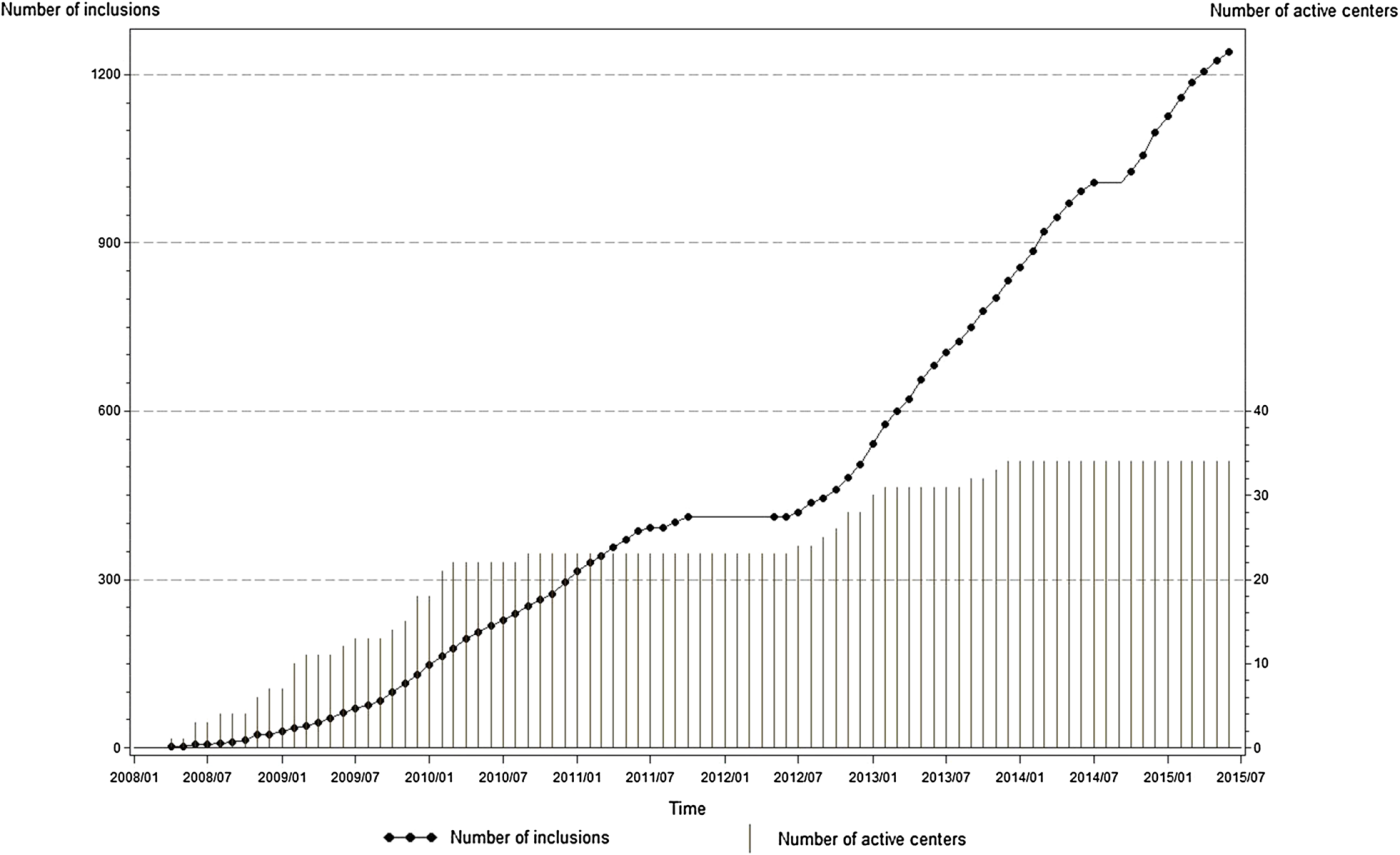
Cumulative number of enrolled patients in the trial

On July 22, 2014, AP-HP decided to suspend the trial after DSMB requested careful examination of serious adverse events and checking study drugs quality. Subsequently, detailed description of all serious adverse events was provided for all patients. Meanwhile, independent investigations confirmed study drugs quality. DSMB advised on October 1, 2014, AP-HP to restart the trial. The trial continuation was approved on October 7, 2014 by ANSM and on November 13, 2014 by CPP. The trial was completed by December 23, 2015, after the last patient reached 180 day post-randomization. Figure 1 shows the actual recruitment curve.

In April 2014, ANSM announced a shortage of Synacthen 0.25 in Europe. The trial was not suspended. Investigators and sites’ pharmacists asked to continue sampling for random cortisol measurement whenever Synacthen was not available at their hospital.

### Study protocol amendments

There were 29 amendments to study protocol (Table 2). They were approved by investigators, study statistician, AP-HP, CPP and ANSM.

**Table 2:** List of amendments to the protocol

Amendments number	Changes in protocol	CPP approval date	ANSM approval date	Official version of trial protocol
1	Changes in following exclusion criteria: “surgical procedure in the past 7 days” was changed for “surgical procedure within 72 h, or any surgery associated with high risk of bleeding, or a planned surgery within 24 h” “chronic liver disease” was clarified as “chronic liver disease with Child score C” “severe thrombopenia” was clarified” as severe thrombopenia (<30,000/mm^3^, before transfusion) Minor wording modifications in title, study acronym, randomization paragraph Recruitment of 4 additional centers	14/02/2008	27/03/2008	Protocol V2 dated 28/01/2008
2	Recruitment of 1 additional center Withdrawal of 1 center	12/06/2008	Not applicable	Protocol V2 dated 28/01/2008 unchanged
3	Recruitment of 9 additional centers	03/07/2008	Not applicable	Protocol V2 dated 28/01/2008 unchanged
4	Recruitment of 1 additional center	13/11/2008	Not applicable	Protocol V2 dated 28/01/2008 unchanged
5	The time window for the use of a prepared (i.e., ready for infusion) infusion bag of Xigris and of its placebo was increased from 14 h to 24 h	12/02/2009	26/12/2008	Protocol V3 dated 11/12/2008
6	Recruitment of 1 additional center Change in contact details of 3 centers	15/01/2009	Not applicable	Protocol V3 dated 11/12/2008 unchanged
7	Recruitment of 1 additional center	9/04/2009	Not applicable	Protocol V3 dated 11/12/2008 unchanged
8	Recruitment of 2 additional centers	18/06/2009	Not applicable	Protocol V3 dated 11/12/2008 unchanged
9	The exclusion criteria: surgical procedure within 72 h, or any surgery associated with high risk of bleeding, or a planned surgery within 24 h “was changed for” surgical procedure within 12 h, or any surgery associated with high risk of bleeding	17/09/2009	25/08/2009	Protocol V3.0 dated 20/07/2009
10	Recruitment of 1 additional center	12/11/2009	Not applicable	Protocol V3.0 dated 20/07/2009 unchanged
11	Recruitment of 1 additional center	10/12/2009	Not applicable	Protocol V3.0 dated 20/07/2009 unchanged
12	Recruitment of 1 additional center	11/03/2010	Not applicable	Protocol V3.0 dated 20/07/2009 unchanged
13	Inclusion criteria: admitted to the ICU for <7 days was removed. New exclusion criteria: patients who had a previous episode of sepsis during the same hospital stay Prolongation of the recruitment period Recruitment of 3 additional centers Withdrawal of 11 inactive centers	10/06/2010	11/06/2010	Protocol V4 dated 10/05/2010
14	Withdrawal of 4 inactive centers	09/09/2010	Not applicable	Protocol V4 dated 10/05/2010 unchanged
15	Recruitment of 1 additional center	23/11/2010	Not applicable	Protocol V4 dated 10/05/2010 unchanged
16	Recruitment of 1 additional center Implementation of biobanking: serum, plasma, DNA Change in informed consent sheet	23/11/2010	09/11/2010	Protocol V6 dated 03/11/2010 Informed consent sheet V1.1 dated 26/10/2010
17	Recruitment of 1 additional center	13/10/2010	Not applicable	Protocol V6 dated 03/11/2010 unchanged
18	Recruitment of 7 additional centers	13/01/2011	Not applicable	Protocol V6 dated 03/11/2010 unchanged
19	Recruitment of 1 additional center	7/04/2011	Not applicable	Protocol V6 dated 03/11/2010 unchanged
20	Recruitment of 1 additional center	12/05/2011	Not applicable	Protocol V6 dated 03/11/2010 unchanged
20 BIS	Following the withdrawal of DAA from the market: Study design was modified from a 2 × 2 factorial to a two-parallel groups’ trial Withdrawal of following exclusion criteria: (1) any surgery in the past 12 h, or any surgery associated with high risk of bleeding; (2) chronic liver disease with a Child score C; (3) recent trauma; (4) any intracranial mass, or stroke or head injury in the past 3 months; (5) severe thrombocytopenia (<30,000/mm^3^, before platelet transfusion); (6) formal indication for anticoagulation, or any other condition associated with increased risk of bleeding, as appreciated by the patient’s physician Change in informed consent form	10/02/2012	18/04/2012	Protocol V7 du 30/11/2011 Informed consent sheet V3.0 dated 05/01/2012
21	Prolongation of recruitment period	05/07/2012	Not applicable	Protocol V8 dated 27/06/2012 Informed consent form V3.1 dated 21/09/2012
22	Recruitment of 2 additional centers	13/09/2012	Not applicable	Protocol V9 dated 27/08/2012
23	Recruitment of 2 additional centers Withdrawal of 4 centers	13/12/2012	Not applicable	Protocol V10 dated 04/12/2012
24	Recruitment of 2 additional centers Removal of exclusion criteria related to XIGRIS Withdrawal of 5 centers	21/03/2013	22/03/2013	Protocol V11.0 dated 20/02/2013 Protocol V11.1 dated 18/03/2013
25	Minor edits to the informed consent form upon CPP request	24/04/2014	Not applicable	Protocol V12 du 15/10/2013 Informed consent form V3.2 dated 21 01 2014
26	Suspension of inclusions upon DSMB request Changes in DSMB members	18/09/2014	29/08/2014	Protocol V13 dated 24/07/2014
27	Request to restart inclusion following: (1) demonstration of the quality of study active drugs and placebos (2) advice from DSMB	13/11/2014	07/10/2014	Protocol V13 dated 24/07/2014, unchanged
28	Prolongation of trial duration	11/06/2015	Not applicable	Protocol V14.0 dated 12/05/2015

*CPP* comité de protection des personnes, *ANSM* Agence nationale de sécurité des médicaments et produits de santé

The main substantial modification to the protocol was approved in October 2011, following DAA withdrawal from the market. Two study arms, i.e., active DAA and its placebo, were stopped, and the design was modified to a two parallel groups. As sample size was originally computed to show a 10 % absolute reduction in 90-day mortality between corticosteroids versus placebo-treated patients, or DAA versus placebo-treated patients, there was no need to modify it. DAA-related exclusion criteria were no longer justified and withdrawn (Table 2). This last amendment to the protocol was associated with a significant (*p* < 0.001) rise in recruitment rate into the study. There were 0.42 ± 0.89 (range 0–9, median 0 [0;1]) and 0.89 ± 1.46 (range 0–11, median 0 [0;1]) patients per site per month, before and after study amendment, respectively.

### Study monitoring

The Steering committee had monthly conference calls. The study site coordinator (Raymond Poincaré hospital) provided on-demand 24/7 services via phone calls or emails to advise investigators about patients’ eligibility or any other issues that may have risen between randomization and 180-day follow-up. There were a total of 28 face-to-face investigators meeting in which trial protocol and procedures were systematically recalled and investigators adherence and compliance to them discussed. A monthly electronic newsletter informed investigators on trial conduct and on any new information from sepsis literature.

DSMB met 5 times. DRRC organized data monitoring and quality audits. Baseline characteristics, eligibility criteria, primary outcome and serious adverse events reported in the CRF were systematically checked against original chart for all research participants. In addition, for one-third of study population, all data reported in CRF were validated against patient’s original chart. Serious adverse events and major protocol violations were reported to DRRC, ANSM and CPP.

The study coordinator had quarterly face-to-face meeting with DRRC, AP-HP, independent pharmacists to monitor trial conduct according to highest standard for protection of research participants.

All randomized patients completed follow-up for the primary outcome and 180-day mortality data.

## Discussion

This trial was designed to assess the role of DAA and corticosteroids in adult septic shock. Evaluation of DAA was terminated early owing to its withdrawal from the market by Lilly [34]. We found no evidence for any benefit from DAA [8]. Owing to the lack of interaction between DAA and corticosteroids, the trial continued to allow evaluation of corticosteroids. The study was suspended on request of DSMB to check safety issues. The two trial suspensions had no impact on its conduct, except delaying its completion. There were several amendments to the protocol mainly related to DAA. In particular, after DAA withdrawal from the market, corresponding exclusion criteria were removed and trial design changed for a two-parallel groups’ design. This amendment accelerated the recruitment rate into the trial. Then, we will explore any interaction between this change in the protocol and response to corticosteroids.

This trial replicated as much as possible Ger-Inf-05 [10] contrasting with CORTICUS [11] and ADRENAL [35] trials (Tables 1, 2). APROCCHSS trial has investigated the effects of hydrocortisone and fludrocortisone at the same doses, durations and routes of administration than for Ger-Inf-05 [10]. Likewise, it focused on patients with persistent vasopressor dependency and organ failure. Finally, non-responders [16] were an a priori defined subgroup in analyzing corticosteroids effects.

### Risk of bias assessment

First, selection biases were minimized. Allocating interventions to research participants used a random list which sequence was computer-generated by an independent statistician. To prevent forth knowledge of forth allocation, randomization was centralized through a secured Web site and used permutation blocks where size was not known by patients, nurses and physicians, investigators, pharmacists, and study sponsor. Thus, the allocation sequence was adequately concealed. Second, to minimize performance biases, we used a centralized procedure for masking corticosteroids and their placebos. Sites pharmacists received sealed boxes containing either active drugs or placebos in identical forms. Blood glucose levels were kept of ≤150 mg/dl. Previous experiences from this group of investigators [10, 36] as from others [11] demonstrated that nurses, physicians and investigators can remain appropriately blinded to corticosteroids administration. Third, to prevent detection biases, for short-term and long-term outcomes, hospital staff, investigators, pharmacists and outcome assessors will remain blinded until public release of trial findings. Finally, there were no obvious attrition biases. There was no lost-to-follow-up for mortality data up to 180 days. According to French regulation, vital status of any citizen is publicly available at city hall of the town the citizen was born.

Other potential sources of bias may include the fact that the study design and statistical analysis plan were reported after all patients have been enrolled. This was done deliberately to also report the way the trial was conducted, in particular any amendment to the protocol. We did not plan any interim analysis and did not perform any. In fact, after the withdrawal of DAA from the market, we reported the analysis of the effects of this drug after checking for the absence of interaction with low-dose steroids. The code was never broken for corticosteroids, and all the parties involved in the trial remained fully blinded. Therefore, it is unlikely that the lack of reporting of the trial protocol before completion of recruitment may be a source of bias.

In conclusion, APROCCHSS trial is the only trial replicating Ger-Inf-05. Its design and conduct allowed appropriate minimization of risk of bias. It will provide sufficient reliable data to inform routine practice for management of adult septic shock. At the present time, the analysis is still pending, and all parties involved in this trial remain blinded.

## Appendix: Study organization

- *Study chairs*: Djillali Annane (Principal Investigator) and Eric Bellissant (Methodologist).
- *Statistics and data management*: Eric Bellissant and Alain Renault.
- *Steering committee*: Djillali Annane (chair), Eric Bellissant, Alain Cariou, Christian Brun Buisson, Claude Martin, Benoit Misset.
- *Monitor*: Valérie Millul: Délégation à la Recherche Clinique, Hôpital Saint-Louis, Paris.
- *Monitoring*: Layide Meaude: URC Paris Ouest, Délégation à la Recherche Clinique, Hôpital Saint-Louis, Paris.
- *Quality Assurance*: Délégation à la Recherche Clinique, Hôpital Saint-Louis, Paris.
- Data safety and Monitoring Board: Gérard Nitenberg (chair) Jacques Bénichou, Didier Dreyfuss.
- *Pharmacists*: Blandine Lehmann: Département Essais Cliniques, AGEPS, AP-HP, Paris.

**Table:** Study centers and investigators

Surname and name of sites principal investigator	Study sites details
Annane, Djillali Fadel, Fouad Polito, Andrea Clair, Bernard Maxime, Virginie Luis, David	Service de Réanimation médicale HOPITAL RAYMOND POINCARE 104 BD RAYMOND POINCARE 92380 GARCHES
Quenot, Jean-Pierre	Service de réanimation 14 RUE GAFFAREL BP 77908 21079 DIJON CEDEX HÔPITAL F. MITTERRAND CHU DE DIJON
Megarbane, Bruno	Service de Réanimation Médicale et Toxicologique Hôpital LARIBOISIERE 2 rue AMBROISE PARE 75010 PARIS
Siami, Shidasp Percheron, Stéphanie	Service d’ Anesthésie-réanimation du Dr. Lorenzo CENTRE HOSPITALIER D’ETAMPES 26 AV CHARLES DE GAULLE 91150 ETAMPES
Mignon, Alexandre Baudin, Francois Antona, Marion Meghenem, Alia Demesmay, Marine	Service d’ Anesthésie Réanimation Hôpital COCHIN 27 R FAUBOURG ST-JACQUES 75014 PARIS
Forceville, Xavier Kuba-Kusuti, Jean-Kamiena Touati, Samia	Service de Réanimation Polyvalente CENTRE HOSPITALIER DE MEAUX 6 R SAINT FIACRE 77100 MEAUX
Reignier, Jean Colin, Gwenhaël Martin-Lefevre, Laurent Bachoumas, Konstantinos Henry-Lagarrigue, Matthieu Yehia, Aihem Lascarrou, Jean-baptiste Lebert, Christine Lacherade, Jean-claude	Service de réanimation CENTRE HOSPITALIER DEPARTEMENTAL SITE DE LA ROCHE-SUR-YONLES OUDAIRIES 85925 LA ROCHE SUR YON CEDEX 9
Asehnoune, Karim Loutrel, Olivier Dumont, Romain Roquilly, Antoine Mahe, Pierre-Joachim Demeure Dit Latte, Dominique Champin, Philippe Arnould, Jean François Cinotti, Raphaël Le Floch, Ronan	Service d’Anesthésie Réanimation chirurgicale, CHU DE NANTES 19 RUE GENERAL MARGUERITTE, 44000 NANTES
Mercier, Emmanuelle Garot, Denis Dequin, Pierre François Perrotin, Dominique Legras, Annick Mankikian, Julie Talec, Patrice Ehrmann, Stephan Joret, Aurélie Lhommet, Claire Joret, Aurélie Lhommet, Claire Rouve, Emmanuelle Bodet-Contentin, Laetitia Jouan, Youenn Salmon-Gandonniere, Charlotte	Service de réanimation polyvalente, CHU BRETONNEAU, 2, BOULEVARD TONNELLE, 37044 TOURS
Antonini, François Martin, Claude Denis Ragonnet, Benoît	Service d’ Anesthésie Réanimation HOPITAL NORD Chemin des BOURRELY 13015 MARSEILLE
Brun Buisson, Christian Razazi, Keyvan De Prost, Nicolas Carteaux, Guillaume	Service de Réanimation Médicale Hôpital HENRI MONDOR 51 AV DE LATTRE DE TASSIGNY 94010 CRETEIL
Wolff, Michel Mourvillier, Bruno Timsit, Jean françois	Service de Réanimation - Maladies Infectieuses Hôpital BICHAT CLAUDE BERNARD 46 Rue HENRI HUCHARD 75018 PARIS
Chagnon, Jean-Luc Ali Ben Ali, Mohamed	Service de Réanimation Polyvalente C.H. DE VALENCIENNES 114 AV DESANDROUINS BP 479 59322 VALENCIENNES
Chimot, Loic Delour, Pierre Dessalles, Pierre Henri Monseau, Yannick Saint-Leger, Mélanie Bedon-Carte, Sandrine	Service de Réanimation CENTRE HOSPITALIER PERIGUEUX 80, AVENUE GEORGES POMPIDOU CS 61205 24019 PERIGUEUX CEDEX
Boulain, Thierry Mathonnet, Armelle Bretagnol, Anne Runge, Isabelle Barbier, François Muller, Gregoire	Service de réanimation polyvalente CHR D’ORLEANS 1, RUE PORTE-MADELEINE – 45000ORLEANS
Francois, Bruno Clavel, Marc Vignon, Philippe Pichon, Nicolas Fedou, Anne-Laure Chapellas, Catherine Galy, Antoine	Service de réanimation polyvalente CHU LIMOGES 2, AVENUE MARTIN LUTHER KING 87042 LIMOGES
Timsit, Jean-Francois	Service de Réanimation médicale CHU DE GRENOBLE Avenue du Maquis du Gresivaudan, Pavillon Dauphiné BP 217 38043 GRENOBLE
Misset, Benoit Garrouste Orgeas, Maité Philippart, François	Service de Réanimation médicale polyvalente HOPITAL SAINT-JOSEPH 185 R RAYMOND LOSSERAND 75674 PARIS
Souweine, Bertrand Ait-Hssain, Ali	Service de Réanimation CHU CLERMONT-FERRAND 58 RUE MONTALEMBERT BP69 63003 CLERMONT FERRAND
Charpentier, Claire	Service de Réanimation chirurgicale HOPITAL CENTRAL 29 AVENUE DU MARECHAL DE LATTRE DE TASSIGNY 54037 NANCY
Mignon, Alexandre Baudin, Francois Antona, Marion Meghenem, Alia Demesmay, Marine	Service d’ Anesthésie Réanimation Hôpital COCHIN 27 R FAUBOURG ST-JACQUES 75014 PARIS
Petitpas, Franck Mimoz, Olivier Nanadoumgar, Hodanou	Service de Réanimation Chirurgicale HÔPITAL DE LA MILETRIE 2 RUE DE LA MILETRIE B. P. 577 86021 POITIERS
Chastre, Jean Combes, Alain Nieszkowska, Ania	Service de Réanimation Médicale C.H.U. PITIE SALPETRIERE 47 BOULEVARD DE L’HOPITAL 75013 PARIS
Amathieu, Roland Levesque, Eric	Réanimations et surveillance continue chirurgicales HOPITAL HENRI MONDOR 51 AV DE LATTRE DE TASSIGNY 94010 CRETEIL
Cook, Fabrice	Réanimations et surveillance continue chirurgicales HOPITAL HENRI MONDOR 51 AV DE LATTRE DE TASSIGNY 94010 CRETEIL
Constantin, Jean-Michel Chartier, Christian Jabaudon, Mathieu Perbet, Sébatien	Service d’ Anesthésie et réanimation HÔPITAL HOTEL DIEU Boulevard Léon Malfreyt 63058 CLERMONT FERRAND CEDEX 1
