# Erratum to: Design and conduct of the activated protein C and corticosteroids for human septic shock (APROCCHSS) trial

**DOI:** 10.1186/s13613-016-0165-1

**Published:** 2016-08-19

**Authors:** Djillali Annane, Christian Brun-Buisson, Alain Cariou, Claude Martin, Benoit Misset, Alain Renault, Blandine Lehmann, Valérie Millul, Virginie Maxime, Eric Bellissant, The APROCCHSS Investigators for the TRIGGERSEP Network

**Affiliations:** 1 General ICU, Service de Réanimation, Hôpital Raymond Poincaré, Laboratory of Infection and Inflammation, U1173, AP-HP, University of Versailles SQY and INSERM, 104 Boulevard Raymond Poincaré, 92380 Garches, France; 2 Service de Réanimation Médicale, Hôpital Henri Mondor, AP-HP, Créteil, France; 3 Service de Réanimation Médicale, Hôpital Cochin, AP-HP, Paris, France; 4 Service d’Anesthésie Réanimation, Hôpital Nord, AP-HM, Marseille, France; 5 Service de Médecine Intensive et Réanimation, Hôpital Saint-Joseph, AP-HP, Université Paris Descartes, Paris Sorbonne Cité, Paris, France; 6 Service de Pharmacologie, Centre d’Investigation Clinique INSERM 1414, CHU de Rennes, Université de Rennes 1, Rennes, France; 7 Département des Essais Cliniques, AGEPS, Paris, France; 8 Délégation à la Recherche Clinique, Hôpital Saint-Louis, AP-HP, Paris, France

## Erratum to: Ann. Intensive Care (2016) 6:43 DOI 10.1186/s13613-016-0147-3

The original version of this article [1] was published with an incomplete version of the table listing “Study Centers and Investigators” found in the Appendix: Study organization.

The full and correct version of this table has been provided below.

**Table:** Study centers and investigators

Surname and name of sites principal investigator	Study sites details
ANNANE, Djillali FADEL Fouad POLITO Andrea CLAIR Bernard MAXIME Virginie LUIS David	Service de Réanimation médicale HOPITAL RAYMOND POINCARE 104 BD RAYMOND POINCARE 92380 GARCHES
QUENOT Jean-Pierre	Service de réanimation 14 RUE GAFFAREL BP 77908 21079 DIJON CEDEX HÔPITAL F. MITTERRAND CHU DE DIJON
MEGARBANE, Bruno	Service de Réanimation Médicale et Toxicologique Hôpital LARIBOISIERE 2 rue AMBROISE PARE 75010 PARIS
SIAMI, Shidasp PERCHERON Stéphanie	Service d’Anesthésie-réanimation du Dr. Lorenzo CENTRE HOSPITALIER D’ETAMPES 26 AV CHARLES DE GAULLE 91150 ETAMPES
CARIOU Alain NGUYEN Yen-Lan DAVIAUD Fabrice BOUGLE Adrien MIRA Jean Paul CHICHE Jean Daniel PENE Frederic MORICHAU-BEAUCHANT Tristan GERI Guillaume	Service de réanimation médicale HOPITAL COCHIN 27 R FAUBOURG ST-JACQUES 75014 PARIS
SCHWEBEL Carole ARA SOMOHANO Claire MINET Clémence LUGOSI Maxime CARTIER Jean Charles POTTON Leïla	Service de Réanimation médicale CHU DE GRENOBLE Avenue du Maquis du Gresivaudan, Pavillon Dauphiné BP 217 38043 GRENOBLE
FORCEVILLE, Xavier KUBA-KUSUTI Jean-Kamiena TOUATI Samia	Service de Réanimation Polyvalente CENTRE HOSPITALIER DE MEAUX 6 R SAINT FIACRE 77100 MEAUX
REIGNIER Jean COLIN Gwenhaël MARTIN-LEFEVRE Laurent BACHOUMAS Konstantinos HENRY-LAGARRIGUE Matthieu YEHIA Aihem LASCARROU Jean-Baptiste LEBERT Christine LACHERADE Jean-Claude	Service de réanimation CENTRE HOSPITALIER DEPARTEMENTAL SITE DE LA ROCHE-SUR-YON LES OUDAIRIES 85925 LA ROCHE SUR YON CEDEX 9
ASEHNOUNE Karim LOUTREL Olivier DUMONT Romain ROQUILLY Antoine MAHE Pierre-Joachim DEMEURE DIT LATTE Dominique CHAMPIN Philippe ARNOULD Jean François CINOTTI Raphaël LE FLOCH Ronan	Service d’Anesthésie Réanimation chirurgicale, CHU DE NANTES 19 RUE GENERAL MARGUERITTE, 44000 NANTES
MERCIER Emmanuelle GAROT Denis DEQUIN Pierre François PERROTIN Dominique LEGRAS Annick MANKIKIAN Julie TALEC Patrice EHRMANN Stephan JORET Aurélie LHOMMET Claire JORET Aurélie LHOMMET Claire ROUVE Emmanuelle BODET-CONTENTIN Laetitia JOUAN Youenn SALMON-GANDONNIERE Charlotte	Service de réanimation polyvalente, CHU BRETONNEAU, 2, BOULEVARD TONNELLE, 37044 TOURS
ANTONINI, François MARTIN Claude Denis RAGONNET Benoît	Service d’ Anesthésie Réanimation HOPITAL NORD Chemin des BOURRELY 13015 MARSEILLE
BRUN BUISSON, Christian RAZAZI Keyvan DE PROST Nicolas CARTEAUX Guillaume	Service de Réanimation Médicale Hôpital HENRI MONDOR 51 AV DE LATTRE DE TASSIGNY 94010 CRETEIL
TIMSIT, Jean François MOURVILLIER Bruno	Service de Réanimation - Maladies Infectieuses Hôpital BICHAT CLAUDE BERNARD 46 Rue HENRI HUCHARD 75018 PARIS
CHAGNON, Jean-Luc ALI BEN ALI Mohamed	Service de Réanimation Polyvalente C.H. DE VALENCIENNES 114 AV DESANDROUINS BP 479 59322 VALENCIENNES
CHIMOT Loic DELOUR Pierre DESSALLES Pierre Henri MONSEAU Yannick SAINT-LEGER Mélanie BEDON-CARTE Sandrine	Service de Réanimation CENTRE HOSPITALIER PERIGUEUX 80, AVENUE GEORGES POMPIDOU CS 61205 24019 PERIGUEUX CEDEX
BOULAIN Thierry MATHONNET Armelle BRETAGNOL Anne RUNGE Isabelle BARBIER François MULLER Gregoire	Service de réanimation polyvalente CHR D’ORLEANS 1, RUE PORTE-MADELEINE – 45000ORLEANS
FRANCOIS Bruno CLAVEL Marc VIGNON Philippe PICHON Nicolas FEDOU Anne-Laure CHAPELLAS Catherine GALY Antoine	Service de réanimation polyvalente CHU LIMOGES 2, AVENUE MARTIN LUTHER KING 87042 LIMOGES
MISSET, Benoit GARROUSTE ORGEAS Maité PHILIPPART François	Service de Réanimation médicale polyvalente HOPITAL SAINT-JOSEPH 185 R RAYMOND LOSSERAND 75674 PARIS
SOUWEINE, Bertrand AIT-HSSAIN Ali	Service de Réanimation CHU CLERMONT-FERRAND 58 RUE MONTALEMBERT BP69 63003 CLERMONT FERRAND
CHARPENTIER, Claire	Service de Réanimation chirurgicale HOPITAL CENTRAL 29 AVENUE DU MARECHAL DE LATTRE DE TASSIGNY 54037 NANCY
MIGNON, Alexandre BAUDIN Francois ANTONA Marion MEGHENEM Alia DEMESMAY Marine	Service d’ Anesthésie Réanimation Hôpital COCHIN 27 R FAUBOURG ST-JACQUES 75014 PARIS
PETITPAS, Franck MIMOZ Olivier NANADOUMGAR Hodanou	Service de Réanimation Chirurgicale HÔPITAL DE LA MILETRIE 2 RUE DE LA MILETRIE B. P. 577 86021 POITIERS
CHASTRE, Jean COMBES Alain NIESZKOWSKA Ania	Service de Réanimation Médicale C.H.U. PITIE SALPETRIERE 47 BOULEVARD DE L’HOPITAL 75013 PARIS
AMATHIEU Roland LEVESQUE Eric	Réanimations et surveillance continue chirurgicales HOPITAL HENRI MONDOR 51 AV DE LATTRE DE TASSIGNY 94010 CRETEIL
COOK Fabrice	Réanimations et surveillance continue chirurgicales HOPITAL HENRI MONDOR 51 AV DE LATTRE DE TASSIGNY 94010 CRETEIL
CONSTANTIN, Jean-Michel CHARTIER Christian JABAUDON Mathieu PERBET Sébatien	Service d’Anesthésie et réanimation HÔPITAL HOTEL DIEU Boulevard Léon Malfreyt 63058 CLERMONT FERRAND CEDEX 1
COLIN, Gwenhaël	Service de Réanimation CENTRE HOSPITALIER RENE DUBOS 6 avenue de l’Ile de France 95303 CERGY PONTOISE CEDEX
RIGAUD Jean-Philippe	Service de Réanimation Polyvalente CENTRE HOSPITALIER DIEPPE AVENUE PASTEUR, BP 219 76200 DIEPPE, FRANCE
HAOUACHE Hakim KAMOUN Walid SLAVOV Velislav	Réanimations et surveillance continue chirurgicales HOPITAL HENRI MONDOR 51 AV DE LATTRE DE TASSIGNY 94010 CRETEIL
LORIFERNE, Jean-François LE FLOCH Anne Sophie	Service de Réanimation HOPITAL SAINT-CAMILLE - BRY S/M 2 R DES PERES CAMILLIENS 94366 BRY SUR MARNE
JOANNES BOYAU, Olivier	Service d’Anesthésie Réanimation HOPITAL DU HAUT LEVEQUE Avenue de Magellan 33604 PESSAC
BRIVET, François	Service de Réanimation Médicale Polyvalente Hôpital ANTOINE BECLERE 157 R DE LA PTE DE TRIVAUX 92140 CLAMART
Ricome, Jean-Louis	Service de Réanimation médico-chirurgicale-SMUR C.H.I POISSY-SAINT GERMAIN 10 Rue du CHAMP GAILLARD BP 3082 78303 POISSY
Bohe, Julien	Service de Réanimation Médicale Sud CENTRE HOSPITALIER LYON-SUD 69495 PIERRE BENITE CEDEX
Slama, Michel	Service de Néphrologie GROUPE HOSPITALIER SUD AV RENE LAENNEC 80054 AMIENS
Leroy, Olivier	Service de Réanimation Médicale et Maladies Infectieuses C.H. TOURCOING GUSTAVE DRON 155 rue DU PRESIDENT COTY BP619 59208 TOURCOING
Capellier, Gilles	Service de Réanimation Médicale - SAMU 25 HOPITAL JEAN MINJOZ 3 BD ALEXANDRE FLEMING 25030 BESANCON
Descorps Desclere, Adrien	Service de Anesthésie Réanimation Chirurgicale Hôpital ANTOINE BECLERE 157 R DE LA PTE DE TRIVAUX 92140 CLAMART
Leon, Alain	Service de Réanimation anesthésie CHU DE REIMS 45 rue Cognacq-Gay 51092 REIMS CEDEX
Mantz, Jean	Service d’Anesthésie Réanimation SMUR Hôpital BEAUJON 100 BD DU GENERAL LECLERC 92110 CLICHY
D’Honneur, Gilles	Service d’Anesthésie Réanimation Hôpital JEAN VERDIER AV DU 14 JUILLET 93140 BONDY
Brenas, François	Service d’Anesthésie-Réanimation CENTRE HOSPITALIER BD DU DR.CHANTEMESSE 43012 LE PUY EN VELAY CEDEX
Benhamou, Michel	Service de Réanimation Cardiaque HOPITAL PRIVE D’ANTONY 1 RUE VELPEAU 92166 ANTONY CEDEX
Starczala, Eric	Service de Réanimation Médicale CENTRE HOSPITALIER LEON BINET DE PROVINS Route de CHALAUTRE 774888 PROVINS CEDEX
BOUGON David	Service de réanimation 1 AVENUE DE L’HOPITAL 74370 EPAGNY METZ-TESSY

We are sorry for any inconvenience this has caused.
